# The Role of Probiotics in the Poultry Industry

**DOI:** 10.3390/ijms10083531

**Published:** 2009-08-12

**Authors:** S. M. Lutful Kabir

**Affiliations:** 1 Graduate School of Life and Environmental Sciences, Osaka Prefecture University, Osaka, Japan; E-Mail: lkabir79@yahoo.com; 2 Department of Microbiology and Hygiene, Faculty of Veterinary Science, Bangladesh Agricultural University, Mymensingh-2202, Bangladesh

**Keywords:** probiotics, bacteria, disease control, meat quality, poultry

## Abstract

The increase of productivity in the poultry industry has been accompanied by various impacts, including emergence of a large variety of pathogens and bacterial resistance. These impacts are in part due to the indiscriminate use of chemotherapeutic agents as a result of management practices in rearing cycles. This review provides a summary of the use of probiotics for prevention of bacterial diseases in poultry, as well as demonstrating the potential role of probiotics in the growth performance and immune response of poultry, safety and wholesomeness of dressed poultry meat evidencing consumer’s protection, with a critical evaluation of results obtained to date.

## 1. Introduction

The poultry industry has become an important economic activity in many countries. In large-scale rearing facilities, where poultry are exposed to stressful conditions, problems related to diseases and deterioration of environmental conditions often occur and result in serious economic losses. Prevention and control of diseases have led during recent decades to a substantial increase in the use of veterinary medicines. However, the utility of antimicrobial agents as a preventive measure has been questioned, given extensive documentation of the evolution of antimicrobial resistance among pathogenic bacteria. So, the possibility of antibiotics ceasing to be used as growth stimulants for poultry and the concern about the side-effects of their use as therapeutic agents has produced a climate in which both consumer and manufacturer are looking for alternatives. Probiotics are being considered to fill this gap and already some farmers are using them in preference to antibiotics [1–3].

Adding the so-called beneficial bacteria to the digestive tract of poultry is not a new concept, however, a complete understanding of where, when and how to use them still has escaped us in its entirety. A strikingly crucial event in the development of probiotics was the finding that newly hatched chickens could be protected against colonization by *Salmonella enteritidis* by dosing a suspension of gut contents derived from healthy adult chickens [4]. This concept is called competitive exclusion.

The impact of biotechnology in poultry nutrition is of significant importance. Biotechnology plays a vital role in the poultry feed industry. Nutritionists are continually putting their efforts into producing better and more economical feed. Good feed alone will not serve the purpose but its better utilization is also essential. Dietary changes as well as lack of a healthy diet can influence the balance of the microflora in the gut thus predisposing to digestion upsets. A well-balanced ration sufficient in energy and nutrients is also of great importance in maintaining a healthy gut. A great deal of attention has recently been received from nutritionists and veterinary experts for proper utilization of nutrients and the use of probiotics for growth promotion of poultry.

In broiler nutrition, probiotic species belonging to *Lactobacillus*, *Streptococcus*, *Bacillus*, *Bifidobacterium*, *Enterococcus*, *Aspergillus*, *Candida*, and *Saccharomyces* have a beneficial effect on broiler performance [5–25], modulation of intestinal microflora and pathogen inhibition [7,20,26–31], intestinal histological changes [29,32,33], immunomodulation [8,10,15,19,22,34–39], certain haemato-biochemical parameters [7,11–12,25,39], improving sensory characteristics of dressed broiler meat [40,41] and promoting microbiological meat quality of broilers [42].

The objectives of this review are to describe the principles, mechanisms of action and criteria for selection of probiotics, and to summarize their applications in the poultry industry.

## 2. What Is a Probiotic?

Over the years the word probiotic has been used in several different ways. It was originally used to describe substances produced by one protozoan which stimulated by another [43], but it was later used to describe animal feed supplements which had a beneficial effect on the host animal by affecting its gut flora [44]. Crawford [45] defined probiotics as “a culture of specific living micro-organisms (primarily *Lactobacillus spp.*) which implants in the animal to ensure the effective establishment of intestinal populations of both beneficial and pathogenic organisms”. Fuller [46] later gave a unique definition of probiotics as “a live microbial feed supplement which beneficially affects the host animal by improving its intestinal microbial balance”. The US National Food Ingredient Association presented, probiotic (direct fed microbial) as a source of live naturally occurring microorganisms and this includes bacteria, fungi and yeast [47]. According to the currently adopted definition by FAO/WHO, probiotics are: “live microorganisms which when administered in adequate amounts confer a health benefit on the host” [48]. More precisely, probiotics are live microorganisms of nonpathogenic and nontoxic in nature, which when administered through the digestive route, are favorable to the host’s health [49].

**Figure 1.**
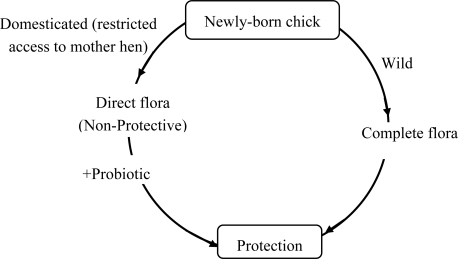
Schematic representation of the concept of probiotics (modified from [50]).

It is believed by most investigators that there is an unsteady balance of beneficial and non-beneficial bacteria in the tract of normal, healthy, non-stressed poultry. When a balance exists, the bird performs to its maximum efficiency, but if stress is imposed, the beneficial flora, especially lactobacilli, have a tendency to decrease in numbers and an overgrowth of the non-beneficial ones seems to occur. This occurrence may predispose frank disease, *i.e.,* diarrhea, or be subclinical and reduce production parameters of growth, feed efficiency, etc. The protective flora which establishes itself in the gut is very stable, but it can be influenced by some dietary and environmental factors. The three most important are excessive hygiene, antibiotic therapy and stress. In the wild, the chicken would receive a complete gut flora from its mother's faeces and would consequently be protected against infection (Figure 1). However, commercially reared chickens are hatched in incubators which are clean and do not usually contain organisms commonly found in the chicken gut. There is an effect of shell microbiological contamination which may influence gut microflora characteristics. Moreover, also HCl gastric secretion, which starts at 18 days of incubation, has a deep impact on microflora selection. Therefore, an immediate use of probiotics supplementation at birth is more important and useful in avian species than in other animals. The chicken is an extreme example of a young animal which is deprived of contact with its mother or other adults and which is, therefore, likely to benefit from supplementation with microbial preparations designed to restore the protective gut microflora [50].

The species currently being used in probiotic preparations are varied and many. These are mostly *Lactobacillus bulgaricus, Lactobacillus acidophilus, Lactobacillus casei, Lactobacillus helveticus, Lactobacillus lactis, Lactobacillus salivarius, Lactobacillus plantarum, Streptococcus thermophilus, Enterococcus faecium, Enterococcus faecalis, Bifidobacterium spp.* and *Escherichia coli.* With two exceptions, these are all intestinal strains. The two exceptions, *Lactobacillus bulgaricus* and *Streptococcus thermophilus*, are yoghurt starter organisms [46]. Some other probiotics are microscopic fungi such as strains of yeasts belonging to *Saccharomyces cerevisiae* species [49,51].

## 3. Mechanisms of Action

Enhancement of colonization resistance and/or direct inhibitory effects against pathogens are important factors where probiotics have reduced the incidence and duration of diseases. Probiotic strains have been shown to inhibit pathogenic bacteria both *in vitro* and *in vivo* through several different mechanisms.

The mode of action of probiotics in poultry includes: (i) maintaining normal intestinal microflora by competitive exclusion and antagonism [4,7,27,29,46,52–60]; (ii) altering metabolism by increasing digestive enzyme activity and decreasing bacterial enzyme activity and ammonia production [61–66]; (iii) improving feed intake and digestion [67–74]; and (iv) stimulating the immune system [10,19,22,37–39,75–79].

Probiotic and competitive exclusion approaches have been used as one method to control endemic and zoonotic agents in poultry. In traditional terms, competitive exclusion in poultry has implied the use of naturally occurring intestinal microorganisms in chicks and poults that were ready to be placed in brooder house. Nurmi and Rantala [4] and Rantala and Nurmi [52] first applied the concept when they attempted to control a severe outbreak of *S. infantis* in Finnish broiler flocks. In their studies, it was determined that very low challenge doses of *Salmonella* (1 to 10 cells into the crop) were sufficient to initiate salmonellosis in chickens. Additionally, they determined that it was during the 1^st^ week post-hatch that the chick was most susceptible to *Salmonella* infections. Use of a *Lactobacillus* strain did not produce protection, and this forced them to evaluate an unmanipulated population of intestinal bacteria from adult chickens that were resistant to *S. infantis*. On oral administration of this undefined mixed culture, adult-type resistance to *Salmonella* was achieved. This procedure later became known as the Nurmi or competitive exclusion concept. The competitive exclusion approach of inoculating day-old chicks with an adult microflora successfully demonstrates the impact of the intestinal microbiota on intestinal function and disease resistance [54,57]. Although competitive exclusion fits the definition of probiotics, the competitive exclusion approach instantaneously provides the chick with an adult intestinal microbiota instead of adding one or a few bacterial species to an established microbial population. Inoculating day-old chicks with competitive exclusion cultures or more classical probiotics serves as a nice model for determining the modes of action and efficacy of these microorganisms. Because of the susceptibility of day-old chicks to infection, this practice is also of commercial importance. By using this model, a number of probiotics [7,27,53–56] have been shown to reduce colonization and shedding of *Salmonella* and *Campylobacter*. Competitive exclusion is a very effective measure to protect newly hatched chicks, turkey poults, quails and pheasants and possibly other game birds, too, against *Salmonella* and other enteropathogens [59].

Upon consumption, probiotics deliver many lactic acid bacteria into the gastrointestinal tract. These microorganisms have been reputed to modify the intestinal milieu and to deliver enzymes and other beneficial substances into the intestines [80]. Supplementation of *L. acidophilus* or a mixture of *Lactobacillus* cultures to chickens significantly increased (*P*<0.05) the levels of amylase after 40 d of feeding [65]. This result is similar to the finding of Collington *et al.* [81], who reported that inclusion of a probiotic (a mixture of multiple strains of *Lactobacillus* spp. and *Streptococcus faecium*) resulted in significantly higher carbohydrase enzyme activities in the small intestine of piglets. The lactobacilli colonizing the intestine may secrete the enzyme, thus increasing the intestinal amylase activity [82,83]. It is well established that probiotics alter gastrointestinal pH and flora to favor an increased activity of intestinal enzymes and digestibility of nutrients [67]. The effect of *Aspergillus oryzae* on macronutrients metabolism in laying hens was observed [59], of which findings might be of practical relevance. They postulated that active amylolytic and proteolytic enzymes residing in *Aspergillus oryzae* may influence the digested nutrients. Similarly, it was reported that an increase in the digestibility of dry matter was closely related to the enzymes released by yeast [64]. In addition, probiotics may contribute to the improvement of health status of birds by reducing ammonia production in the intestines [63].

Probiotic is a generic term, and products can contain yeast cells, bacterial cultures, or both that stimulate microorganisms capable of modifying the gastrointestinal environment to favor health status and improve feed efficiency [67]. Mechanisms by which probiotics improve feed conversion efficiency include alteration in intestinal flora, enhancement of growth of nonpathogenic facultative anaerobic and gram positive bacteria forming lactic acid and hydrogen peroxide, suppression of growth of intestinal pathogens, and enhancement of digestion and utilization of nutrients [70]. Therefore, the major outcomes from using probiotics include improvement in growth [70], reduction in mortality [71], and improvement in feed conversion efficiency [70]. These results are consistent with previous experiment of Tortuero and Fernandez [72], who observed improved feed conversion efficiency with the supplementation of probiotic to the diet.

The manipulation of gut microbiota via the administration of probiotics influences the development of the immune response [75]. The exact mechanisms that mediate the immunomodulatory activities of probiotics are not clear. However, it has been shown that probiotics stimulate different subsets of immune system cells to produce cytokines, which in turn play a role in the induction and regulation of the immune response [84–86]. Stimulation of human peripheral blood mononuclear cells with *Lactobacillus rhamnosus* strain GG *in vitro* resulted in the production of interleukin 4 (IL-4), IL-6, IL-10, tumor necrosis factor alpha, and gamma interferon [87]. Other studies have provided confirmatory evidence that Th2 cytokines, such as IL-4 and IL-10, are induced by lactobacilli [84–85,88]. The outcome of the production of Th2 cytokines is the development of B cells and the immunoglobulin isotype switching required for the production of antibodies. The production of the mucosal IgA response is dependent on other cytokines, such as transforming growth factor β [89]. Importantly, various species and strains of lactobacilli are able to induce the production of transforming growth factor β, albeit to various degrees [90]. Probiotics, especially lactobacilli, could modulate the systemic antibody response to antigens in chickens [10,22,37,39,76,77].

## 4. Criteria for Selection of Probiotics in the Poultry Industry

**Figure 2.**
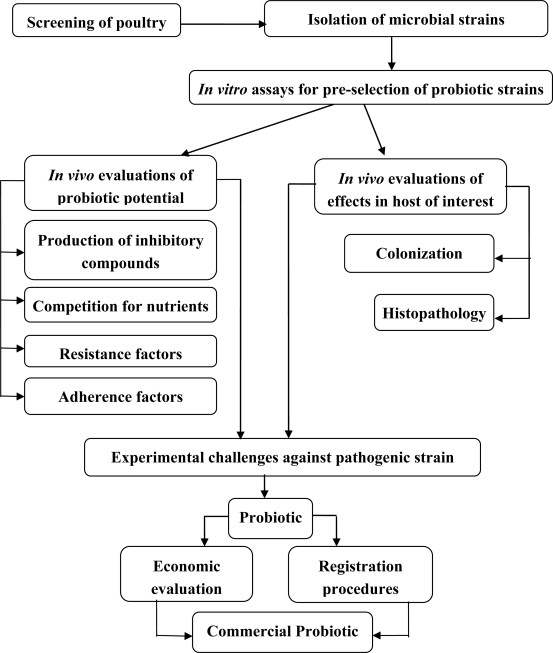
Diagram for selection of probiotics in the poultry industry (modified from [93–97]).

The perceived desirable traits for selection of functional probiotics are many. The probiotic bacteria must fulfill the following conditions: it must be a normal inhabitant of the gut, and it must be able to adhere to the intestinal epithelium to overcome potential hurdles, such as the low pH of the stomach, the presence of bile acids in the intestines, and the competition against other micro-organisms in the gastro-intestinal tract [91,92]. The tentative ways for selection of probiotics as biocontrol agents in the poultry industry are illustrated in Figure 2. Many *in vitro* assays have been developed for the pre-selection of probiotic strains [93–95]. The competitiveness of the most promising strains selected by *in vitro* assays was evaluated *in vivo* for monitoring of their persistence in chickens [96]. In addition, potential probiotics must exert its beneficial effects (*e.g.,* enhanced nutrition and increased immune response) in the host. Finally, the probiotic must be viable under normal storage conditions and technologically suitable for industrial processes (*e.g.,* lyophilized).

## 5. Evaluating Probiotic Effects on Growth Performance

Studies on the beneficial impact on poultry performance have indicated that probiotic supplementation can have positive effects. It is clearly evident from the result of Kabir *et al.* [10] that the live weight gains were significantly (*P*<0.01) higher in experimental birds as compared to control ones at all levels during the period of 2^nd^, 4^th^, 5^th^ and 6^th^ weeks of age, both in vaccinated and nonvaccinated birds. This result is in agreement with many investigators [7–9,11–25] who demonstrated increased live weight gain in probiotic fed birds. On the other hand, Lan *et al.* [98] found higher (*P*<0.01) weight gains in broilers subjected to two probiotic species. Huang *et al.* [76] demonstrated that inactivated probiotics, disrupted by a high-pressure homogenizer, have positive effects on the production performance of broiler chickens when used at certain concentrations. In addition, Torres-Rodriguez *et al*. [99] reported that administration of the selected probiotic (FM-B11) to turkeys increased the average daily gain and market BW, representing an economic alternative to improve turkey production. However, Karaoglu and Durdag [100] used *Saccharomyces cerevisiae* as a dietary probiotic to assess performance and found no overall weight gain difference.

Kabir *et al.* [10] reported the occurrence of a significantly (*P*<0.01) higher carcass yield in broiler chicks fed with the probiotics on the 2^nd^, 4^th^ and 6^th^ week of age both in vaccinated and nonvaccinated birds. Although Mahajan *et al.* [101] recorded in their study that mean values of giblets, hot dress weight, cold dress weight and dressing percentage were significantly (*P*<0.05) higher for probiotic (Lacto-Sacc) fed broilers. On the other hand, Mutus *et al.* [102] investigated the effects of a dietary supplemental probiotic on morphometric parameters and yield stress of the tibia and they found that tibiotarsi weight, length, and weight/length index, robusticity index, diaphysis diameter, modulus of elasticity, yield stress parameters, and percentage Ca content were not affected by the dietary supplementation of probiotic, whereas thickness of the medial and lateral wall of the tibia, tibiotarsal index, percentage ash, and P content were significantly improved by the probiotic.

## 6. Evaluating Probiotic Effects on the Intestinal Microbiota and Intestinal Morphology

Kabir *et al.* [29] attempted to evaluate the effect of probiotics with regard to clearing bacterial infections and regulating intestinal flora by determining the total viable count (TVC) and total lactobacillus count (TLC) of the crop and cecum samples of probiotics and conventional fed groups at the 2nd, 4th and 6th week of age. Their result revealed competitive antagonism. The result of their study also evidenced that probiotic organisms inhibited some nonbeneficial pathogens by occupying intestinal wall space. They also demonstrated that broilers fed with probiotics had a tendency to display pronounced intestinal histological changes such as active impetus in cell mitosis and increased nuclear size of cells, than the controls. This results of histological changes support the findings of Samanya and Yamauchi [32] and they indicated that birds who were fed dietary *B. subtilis* var. natto for 28 days had a tendency to display greater growth performance and pronounced intestinal histologies, such as prominent villus height, extended cell area and consistent cell mitosis, than the controls. On the other hand, Chichlowski *et al.* [33] compared the effects of providing a direct-fed microbials (DFM) with the feeding of salinomycin on intestinal histomorphometrics, and microarchitecture and they found less mucous thickness in DFM-treated chickens and the density of bacteria embedded in the mucous blanket appeared to be lower in DFM-treated chickens than in the control in all intestinal segments. Watkins and Kratzer [103] reported that chicks dosed with *Lactobacillus* strains had lower numbers of coliforms in cecal macerates than the control. Francis *et al*. [104] also reported that the addition of *Lactobacillus* product at 75 mg/kg of feed significantly decreased the coliform counts in the ceca and small intestine of turkeys. Using gnotobiotic chicks, Fuller [105] found that host-specific *Lactobacillus* strains were able to decrease *Escherichia coli* in the crop and small intestine. Kizerwetter-Swida and Binek [60] demonstrated that *L. salivarius* 3d strain reduced the number of *Salmonella enteritidis* and *Clostridium perfringens* in the group of chickens treated with Lactobacillus. Watkins *et al*. [106] similarly observed that competitive exclusion of pathogenic *E. coli* occurred in the gastrointestinal tract of gnotobiotic chicks dosed with *L. acidophilus*. Recently Yaman *et al.* [30]; Mountzouris *et al.* [20] and Higgins *et al.* [31] demonstrated that probiotic species belonging to *Lactobacillus*, *Streptococcus*, *Bacillus*, *Bifidobacterium*, *Enterococcus*, *Aspergillus*, *Candida*, and *Saccharomyces* have a potential effect on modulation of intestinal microflora and pathogen inhibition.

## 7. Evaluating Probiotic Effects on Immune Response

Kabir *et al.* [10] evaluated the dynamics of probiotics on immune response of broilers and they reported significantly higher antibody production (*P*<0.01) in experimental birds as compared to control ones. They also demonstrated that the differences in the weight of spleen and bursa of probiotics and conventional fed broilers could be attributed to different level of antibody production in response to SRBC. Similarly, Khaksefidi and Ghoorchi [15] reported that the antibody titer in the 50 mg/kg probiotic supplemented group was significantly higher at 5 and 10 days of postimmunization (PI) compared to control, when SRBC was injected at 7 and 14 days of age. In addition, Haghighi *et al.* [37] demonstrated that administration of probiotics enhances serum and intestinal natural antibodies to several foreign antigens in chickens. On the other hand, Dalloul *et al.* [78] examined the effects of feeding a *Lactobacillus*-based probiotic on the intestinal immune responses of broiler chickens over the course of an *E. acervulina* infection and they demonstrated that the probiotic continued to afford some measure of protection through immune modulation despite a fairly overwhelming dose of *E. acervulina*. They also suggested a positive impact of the probiotic in stimulating some of the early immune responses against *E. acervulina*, as characterized by early IFN-γ and IL-2 secretions, resulting in improved local immune defenses against coccidiosis. Brisbin *et al.* [79] investigated spatial and temporal expression of immune system genes in chicken cecal tonsil and spleen mononuclear cells in response to structural constituents of *L. acidophilus* and they found that cecal tonsil cells responded more rapidly than spleen cells to the bacterial stimuli, with the most potent stimulus for cecal tonsil cells being DNA and for splenocytes being the bacterial cell wall components. They also discovered that in both splenocytes and cecal tonsil cells, STAT2 and STAT4 genes were highly induced and the expression of STAT2, STAT4, IL-18, MyD88, IFN-alpha, and IFN-gamma genes were up-regulated in cecal tonsil cells after treatment with *L. acidophilus* DNA. Simultaneously, several investigators demonstrated the potential effect of probiotic on immunomodulation [34,8,35–37,39,19,22]. On the other hand, Midilli *et al.* [107] showed the ineffectiveness of additive supplementation of probiotics on systemic IgG.

## 8. Evaluating Probiotic Effects on Meat Quality

Kabir [40] and Kabir *et al.* [42] evaluated the effects of probiotics on the sensory characteristics and microbiological quality of dressed broiler meat and reported that supplementation of probiotics in broiler ration improved the meat quality both at prefreezing and postfreezing storage. Mahajan *et al.* [108] stated that the scores for the sensory attributes of the meat balls appearance, texture, juiciness and overall acceptability were significantly (p60.001) higher and those for flavour were lower in the probiotic (Lacto-Sacc) fed group. Simultaneously, Mahajan *et al.* [108] reported that meat from probiotic (Lacto-Sacc) fed birds showed lower total viable count as compared to the meat obtained from control birds. On the other hand, Loddi *et al.* [109] reported that neither probiotic nor antibiotic affected sensory characteristics (intensity of aroma, strange aroma, flavour, strange flavour, tenderness, juiciness, acceptability, characteristic colour and overall aspects) of breast and leg meats. On the other hand, Zhang *et al.* [110] conducted an experiment with 240, day-old, male broilers to investigate the effects of *Saccharomyces cerevisiae* (SC) cell components on the meat quality and they reported that meat tenderness could be improved by the whole yeast (WY) or *Saccharomyces cerevisiae* extract (YE).

## 9. Conclusions

The concept of probiotics in recent year is no more confusing as was earlier thought. It now constitutes an important aspect of applied biotechnological research and therefore as opposed to antibiotics and chemotherapeutic agents can be employed for growth promotion in poultry. In past years, men considered all bacteria as harmful, forgetting about the use of the organisms in food preparation and preservation, thus making probiotic concept somewhat difficult to accept. Scientists now are triggering effort to establish the delicate symbiotic relationship of poultry with their bacteria, especially in the digestive tract, where they are very important to the well being of man and poultry. Since probiotics do not result in the development and spread of microbial resistance, they offer immense potential to become an alternative to antibiotics. The present review reveals that probiotics could be successfully used as nutritional tools in poultry feeds for promotion of growth, modulation of intestinal microflora and pathogen inhibition, immunomodulation and promoting meat quality of poultry.
